# Copper pyrithione and zinc pyrithione induce cytotoxicity and neurotoxicity in neuronal/astrocytic co-cultured cells via oxidative stress

**DOI:** 10.1038/s41598-023-49740-8

**Published:** 2023-12-27

**Authors:** Ha-Na Oh, Woo-Keun Kim

**Affiliations:** 1 Department of Predictive Toxicology, Korea Institute of Toxicology, Daejeon, 34114 Republic of Korea; 2 Human and Environmental Toxicology, University of Science and Technology, Daejeon, 34113 Republic of Korea

**Keywords:** Cell biology, Neuroscience, Environmental sciences

## Abstract

Previous studies on copper pyrithione (CPT) and zinc pyrithione (ZPT) as antifouling agents have mainly focused on marine organisms. Even though CPT and ZPT pose a risk of human exposure, their neurotoxic effects remain to be elucidated. Therefore, in this study, the cytotoxicity and neurotoxicity of CPT and ZPT were evaluated after the exposure of human SH-SY5Y/astrocytic co-cultured cells to them. The results showed that, in a co-culture model, CPT and ZPT induced cytotoxicity in a dose-dependent manner (~ 400 nM). Exposure to CPT and ZPT suppressed all parameters in the neurite outgrowth assays, including neurite length. In particular, exposure led to neurotoxicity at concentrations with low or no cytotoxicity (~ 200 nM). It also downregulated the expression of genes involved in neurodevelopment and maturation and upregulated astrocyte markers. Moreover, CPT and ZPT induced mitochondrial dysfunction and promoted the generation of reactive oxygen species. Notably,* N*-acetylcysteine treatment showed neuroprotective effects against CPT- and ZPT-mediated toxicity. We concluded that oxidative stress was the major mechanism underlying CPT- and ZPT-induced toxicity in the co-cultured cells.

## Introduction

Antifouling agents are used to inhibit the growth of marine organisms on submerged structures, such as ship hulls, owing to their biocidal action. Tributyltin, a biocide that has been widely used since the early 1970s, has been restricted worldwide because of its harmful effects on the marine environment, and alternative antifouling agents have been developed^1^. Currently, copper pyrithione (CPT) and zinc pyrithione (ZPT) are biocides that are commonly used as antibacterial, antifungal, and antifouling agents^2^. Along with household chemicals and fragrances, CPT and ZPT are commonly found in biocide products and have been reported to be part of a mixture of frequently used biocides^3^. ZPT is also found in skin-hygiene products^4^. Because these substances are used for various purposes other than antifouling, the risk of human exposure to them is high. The acceptable exposure level for CPT and ZPT was determined to be 0.005 mg/kg bw/day, with an overall no-observed-adverse-effect level reported as 0.005^5^. The precise extent of human exposure to CPT remains unknown, and exposure levels may vary depending on the work environment, methods employed, and the use of personal protective equipment. The oral absorption rate of CPT was estimated to be 80–90% in human models, whereas the dermal absorption rate was approximately 3%^5^. Studies have revealed that the level of ZPT deposited on the scalp and hand skin after repeated shampoo use can reach up to 2.6 mg^6^. Furthermore, when ZPT is topically applied, the zinc concentration increases up to 250-fold after 48 h^6^. Human exposure to CPT and ZPT primarily occurs via the dermal or inhalation routes and poses a potential risk to vulnerable groups such as pregnant women, fetuses, children, and the elderly^3^. CPT and ZPT toxicity risk has been further evaluated using an in silico model for biocidal substances that demonstrated that these substances could penetrate the blood–brain barrier (BBB)^7^. In contrast, analysis using the SwisADME tool demonstrated that CPT and ZPT cannot cross the BBB^8,9^ and are instead metabolized into several metabolites^10,11^. The conflicting results obtained with the two predictive models are concerning, but the potential for neurotoxicity cannot be ruled out as ZPT has been reported to induce neuropathy in rats^12^. CPT and ZPT exposure in marine fish has also recently been reported to induce developmental toxicity and affect nervous system development ^10,13,15^. As antifouling agents, CPT and ZPT can be photodegraded to less toxic compounds within minutes; however, in low-light situations or at night, the compounds can accumulate in sediments and exhibit toxic effects^13^. The toxicity of CPT and ZPT has been primarily evaluated using various aquatic organisms, and most of them have exhibited high toxicity^1,14,15^. Accumulating chemicals in marine ecosystems can harm several aquatic organisms and, in turn, humans. The toxicity of ZPT has been reported in several mammalian cells^16–18^, whereas that of CPT remains unknown. Moreover, although ZPT has demonstrated toxicity in human skin fibroblasts^16^, cervical tumor cells^18^, and lymphocytes^19^, its effect on human neurons remains to be evaluated.

Biological, physical, or chemical agents (e.g., industrial chemicals, pharmaceuticals, and naturally-occurring substances) that induce changes in the structure or function of the nervous system can be considered neurotoxic. Representative neurotoxicity testing guidelines require a substantial number of animals and considerable time^20^. Therefore, the use of in vitro cell culture systems prior to in vivo studies can be a more time- and cost-efficient approach. The human-derived SH-SY5Y cell line, which can be differentiated to express neuronal properties, has been used as a model in several studies for evaluating neurotoxicity^21,22^. SH-SY5Y cells are readily differentiated by retinoic acid (RA) and acquire neuron-like properties, including extensive neurite outgrowth and increased levels of neuron-associated proteins^21^. Neurodevelopmental processes can be analyzed using in vitro systems, and neurite outgrowth assays are primarily used to assess the effects of chemicals on neurodevelopment^23^.

In an in vitro neurotoxicity test system, the neuron and astrocyte co-culture model can more closely represent the structure and function of human central nervous system (CNS), as compared with the neuronal model alone^24,25^. Astrocytes regulate synaptic transmission via direct contact with neurons and are involved in the overall activity of neuronal circuits^26^. Recent co-culture studies have explored various cellular responses by creating more biologically relevant conditions using 3D co-culture systems or triple co-culture systems^27,28^. However, 2D co-culture models offer distinct advantages by providing a simple and controlled platform for studying cell interactions, signal transduction pathways, and responses to stimuli. Direct neuron/astrocyte co-culture plays a crucial role in neuronal differentiation and development via complex interactions rather than simple attachment^29^. A recent study reported the investigation of similar aspects of interest such as reactive oxygen species (ROS) generation and toxicity with a co-culture model utilizing neurons and astrocytes, as examined in this study^30^. Our previous study has revealed that the neurotoxic effects of chemicals on a neuron/astrocyte co-culture model differ from those on the neurons alone^25^. Astrocytes play a principal role in maintaining brain homeostasis and have intrinsic neuroprotective properties; thus, the interaction between neurons and astrocytes in a co-culture model can exhibit cytoprotective effects against neurotoxins^29,31^. Nevertheless, cellular dysfunction induced by various internal and external stimuli can ultimately impair neuronal function and viability.

Oxidative stress, a key factor in neurotoxicity development, is caused by the excessive production of free radicals due to insufficient antioxidant defense mechanisms^32^. Adequate levels of ROS are essential for neuronal development and function; however, excessive ROS levels induced by various stimuli lead to neurotoxicity^33^. ROS can induce neuronal cell death by increasing the BBB permeability, and the concomitant mitochondrial impairment ultimately adversely affects the brain^32^. Mitochondria can produce ROS; however, excessive ROS can cause mitochondrial dysfunction^33,34^. The mitochondrial bioenergetic mechanism is closely related to neuronal survival and death^34^.* N*-acetylcysteine (NAC), a precursor of glutathione, is a well-known antioxidant that effectively reduces oxidative stress and has been extensively used to protect against the toxic effects of chemicals in several cell models^18,35–37^. In in vitro as well as in vivo models and preliminary clinical trials, NAC has been reported to attenuate neurotoxicity and exhibit positive impacts on dopamine function and clinical symptoms^38,39^

The aim of this study was to evaluate the potential cytotoxic and neurotoxic effects of CPT and ZPT on neuron/astrocyte co-culture models and elucidate the underlying molecular mechanisms. These results are useful for predicting potential neurotoxicity induced in humans and can serve as a fundamental basis for the use and handling of antifouling agents.

## Material and methods

### Materials and chemicals

Dulbecco’s modified Eagle’s medium (DMEM), fetal bovine serum (FBS), N-2 supplement, trypsin/EDTA, and penicillin/streptomycin (P/S) were purchased from Gibco (Thermo Fisher Scientific, Inc., Waltham, MA, USA). Matrigel and phosphate-buffered saline (PBS) were obtained from Corning Inc. (Corning, NY, USA). CPT (CAS No. 14915-37-8) was obtained from Biosynth Carbosynth (Compton, UK). ZPT (CAS No. 13463-41-7). NAC, RA, and dimethyl sulfoxide (DMSO) were purchased from Sigma-Aldrich (St. Louis, MO, USA).

### Chemical preparation and exposure

CPT and ZPT stock solutions were dissolved in DMSO at a 100 mM concentration. After a 5-day differentiation of the co-cultured cells, they were exposed to various concentrations of CPT or ZPT—100, 150, 200, 300, and 400 nM—for 24 h. As a vehicle control, the final concentration of DMSO was ≤ 0.1%. Simultaneously, a NAC stock solution was prepared in PBS at a 1 M concentration. Cells were pretreated with NAC a concentration of 2 mM for 1 h before exposure to CPT and ZPT.

### Cell culture

Human-induced pluripotent stem cell-derived iCell astrocytes were purchased from the Cellular Dynamics International (ASC-100-020-001-PT; Madison, WI, USA). iCell astrocytes were cultured in DMEM containing 10% heat-inactivated FBS, 1% P/S, and 1% N-2 supplement, and the culture surfaces were pre-coated with Matrigel. SH-SY5Y cells were obtained from the Korea Cell Line Bank (Seoul, Republic of Korea) and cultured in DMEM supplemented with 10% FBS and 1% P/S. Differentiation of SH-SY5Y cells to neurons was established by adding RA to the medium (DMEM containing 1% FBS). Both cell lines were incubated in a humidified 5% CO_2_ atmosphere at 37 °C. The culture medium was replaced every 2–3 days.

### Neuron and astrocyte co-culture

The co-culture system was established according to Oh et al*.*^25^. Astrocyte cells were plated on Matrigel-coated 96- or 48-well plates at a density of 2 × 10^3^/well for 24 h. SH-SY5Y neurons were plated onto the astrocyte monolayer at 1 × 10^4^/well. Astrocytes and SH-SY5Y cells were co-cultured at a ratio of 1:5 in complete DMEM. Twenty-four hours after seeding, the medium was replaced with DMEM containing 1% FBS and 10 μM RA. The differentiation medium was refreshed every three days, and the total differentiation period was five days.

### Western blotting

Cell extracts were harvested using 1X RIPA lysis buffer (Rockland Immunochemicals, Inc., Royersford, PA, USA) containing protease and phosphatase inhibitor cocktails (Cell Signaling Technology, Inc., Danvers, MA, USA). Protein concentration was quantified using a Pierce BCA Protein Assay Kit (Thermo Fisher Scientific, Inc.) as per the manufacturer’s protocol. Equal amounts of protein were separated using 8–15% sodium dodecyl sulfate–polyacrylamide gel electrophoresis and transferred to polyvinylidene fluoride membranes. The polyvinylidene fluoride membranes were blocked in tris-buffered saline buffer with 0.1% Tween 20 containing 3% or 5% skim milk for 1 h at room temperature (RT), and incubated with 1:1000 dilution of primary antibodies at 4 °C overnight, followed by incubation with secondary antibodies at a 1:4000 dilution for 2 h at RT. WesternBright ECL (Advansta Inc., San Jose, CA, USA) was used for protein detection, and ChemiDoc XRS+ imaging system (Bio-Rad Laboratories Inc., Hercules, CA, USA) was used for protein band imaging. Uncropped protein band images are depicted in Supplementary Fig. 1.

### MTS assay

Proliferation of the cells cultured in the 96-well plates was quantified using the CellTiter 96 Aqueous One Solution Cell Proliferation Assay (MTS Assay; Promega Corp., Madison, WI, USA). Briefly, 20 μl of MTS solution was added to each well and the plate was incubated at 37 °C for 2 h. Absorbance was measured at 490 nm using a Cytation 5 microplate reader (Agilent Technologies, Inc., Santa Clara, CA, USA).

### Lactate dehydrogenase (LDH) assay

Cytotoxicity was evaluated using a CytoTox 96 Non-Radioactive Cytotoxicity Assay Kit (Promega Corp.), according to the manufacturer’s instructions. Briefly, cells were seeded in 96-well plates and exposed to CPT and ZPT for 24 h. Cell culture supernatants were transferred to a new plate and reacted with the CytoTox reagent at RT for 30 min. The absorbance was determined at 490 nm using a microplate reader (Cytation 5) to quantify LDH release. Cytotoxicity was represented as a percentage versus maximum LDH value (cell lysate).

### Immunocytochemistry

**Figure 1:**
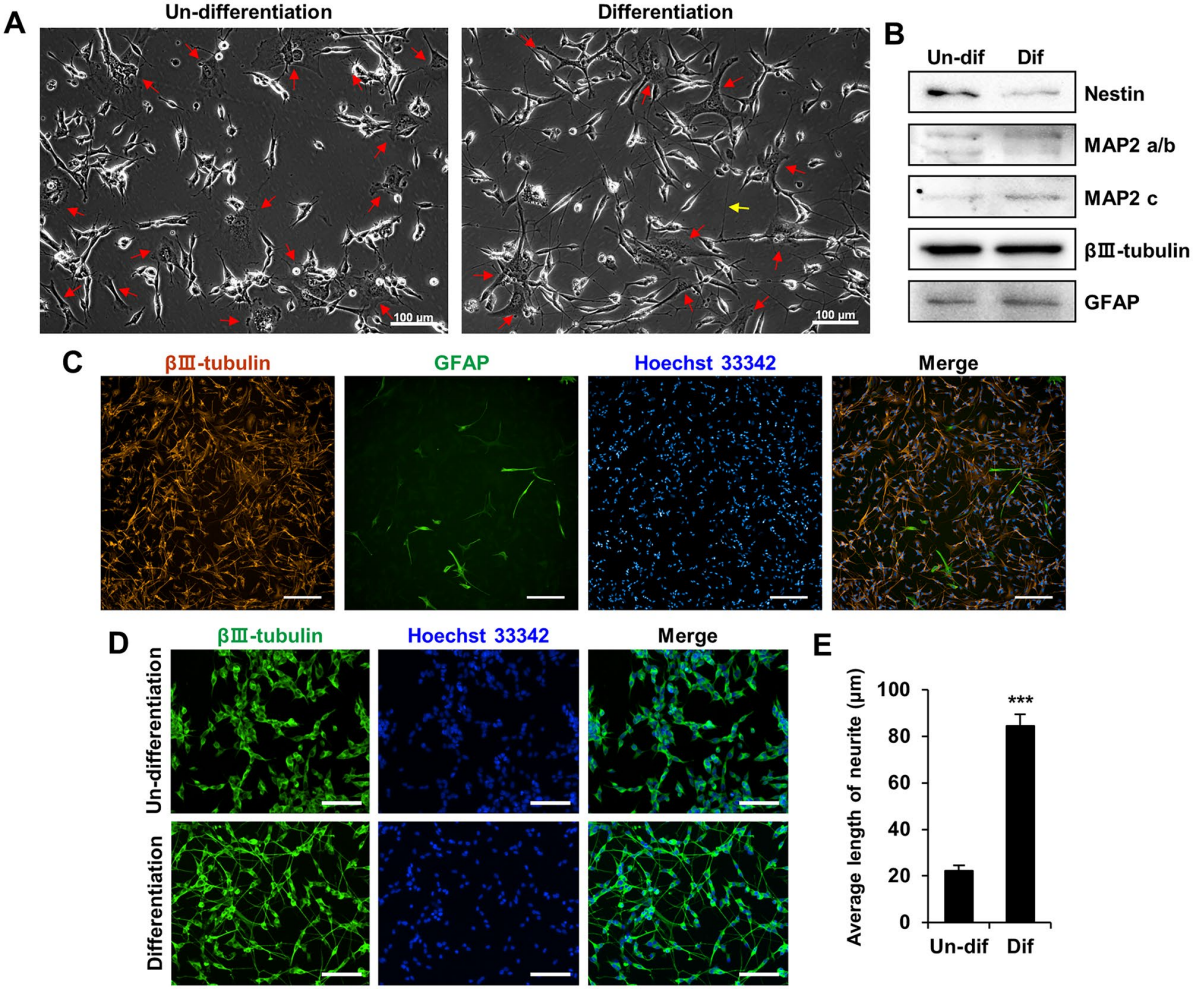
Morphological changes caused by retinoic acid (RA) in a human co-culture cell model. (**A**) Images of the co-cultured cells captured by phase-contrast microscopy: (left) undifferentiated cells in complete medium, (right) differentiated cells in low serum medium supplemented with RA for 5 days. Scale bar: 100 μm. Red arrows indicate astrocytes. Yellow arrows show some neurites of SH-SY5Y cells. (**B**) Neuronal markers (nestin, microtubule-associated protein 2 and βIII-tubulin) and astrocyte marker (GFAP) expression as determined by western blot analysis. (**C**) The images were acquired using a fluorescence microscope. Cells were co-stained with an anti-βIII-tubulin antibody (orange) and an anti-GFAP antibody (green) in the presence of RA. Nuclei were counterstained with Hoechst 33342 (blue). Scale bar: 200 μm. (**D**) The images were obtained using a fluorescence microscope with a 10× objective lens. Cells were stained with an anti-βIII-tubulin antibody (green) in presence and absence of RA. Nuclei were counterstained with Hoechst 33342 (blue). Scale bar: 100 μm. (**E**) Bar graph displays the average length of neurites following the treatments with RA for 5 days. ***p < 0.001.

At the end of the 24 h exposure periods, the plates were washed with 1X PBS, fixed with 4% paraformaldehyde in PBS for 10 min, and then rinsed with PBS. The cells were permeabilized with 0.1% Triton X-100 in PBS for 10 min, and the plates were incubated with a blocking solution containing 0.1% bovine serum albumin, 10% FBS, and 1X PBS for 1 h at RT. As shown in Fig. 1C, neuronal cells were labeled with anti-βIII-tubulin, and astrocytes were labeled with anti-glial fibrillary acidic protein (GFAP) primary antibody, followed by Alexa Fluor 594 and 488-conjugated secondary antibodies, respectively (Abcam, Waltham, MA, USA). The nuclei were counterstained with Hoechst 33342 (Invitrogen, Thermo Fisher Scientific, Inc.). After rinsing with 1X PBS, the plates were stored in PBS at 4 °C until observation.

### Assessment of neurite outgrowth

Neurite outgrowth was evaluated using the ImageXpress Micro XLS Widefield High-Content Screening System (Molecular Devices, LLC., San Jose CA, USA) with a 10× objective. This system automatically focuses on and scans the fields of individual wells. Fluorescence images were obtained using a high-resolution camera and matched excitation filters for channels 1 (blue; Hoechst 33342) and 2 (green; βIII-tubulin). The measured object was counted as a neuron cell if it had valid measurements, including size, shape, and intensity. The resulting fluorescence images were analyzed to obtain various parameters, such as total neurite outgrowth, total nuclei, total processes, and total branches, using the MetaXpress High-Content Image Acquisition and Analysis Software (Molecular Devices, LLC.).

### RNA extraction and relative quantitative real-time polymerase chain reaction (qRT-PCR) analysis

Total RNA was isolated from cells using the Monarch Total RNA Miniprep Kit (New England Biolabs, Ipswich, MA, USA), and the total RNA concentration was determined using a NanoDrop ND-1000 spectrophotometer (Thermo Fisher Scientific, Inc.). The iScript cDNA synthesis Kit (Bio-Rad Laboratories, Inc.) was used to synthesize cDNA from 1 µg total RNA. The cDNA samples were analyzed by qRT-PCR using the SYBR Green PCR Master Mix (Promega Corp.) on a QuantStudio 3 system (Applied Biosystems, Thermo Fisher Scientific, Inc.). The amplification conditions were pre-incubation at 95 °C for 10 min, followed by 40 cycles of 95 °C for 10 s, 60 °C for 20 s, and 72 °C for 20 s. Gene expression was calculated using the comparative CT method (2^−ΔΔCT^)^40^ and normalized against the endogenous control gene *β-actin*. The gene-specific primers used are listed in Table 1.

**Table 1:** List of qRT-PCR primers.

Gene	Forward primer	Reverse primer
*NES*	AACAGCGACGGAGGTCTCTA	TTCTCTTGTCCCGCAGACTT
*TUBB3*	CATCCAGAGCAAGAACAGCA	CTCGGTGAACTCCATCTCGT
*MAP2*	TGCCATCTTGGTGCCGA	CTTGACATTACCACCTCCAGGT
*GAP43*	AGGGAGAAGGCACCACTACT	GGAGGACGGCGAGTTATCAG
*RET*	TTGCCCAGATCGGGAAAGTC	GGCCACCACCATGTAGTGAA
*GFAP*	GAGGTAAGGAGGGATTTGGGC	TGGAGGGACTGAGGAAACGG
*S100β*	AGCTGCCCTGAAGGTGATTTC	CCACACTGAGGGTTCCGTT
*β-actin*	CATGTACGTTGCTATCCAGGC	CTCCTTAATGTCACGCACGAT

### Measurement of ROS

Intracellular ROS were detected using a DCFDA/H2DCFDA-Cellular ROS Assay Kit (Abcam) according to the manufacturer’s protocol. After treatment, the cells were incubated with 1X DCFDA solution for 45 min at 37 °C in the dark and washed with Dulbecco’s PBS. Then, the plates were incubated with 1X supplemented buffer (1X buffer with 1% FBS) and tert-butyl hydroperoxide solution (positive control) for 1 h at 37 °C. The fluorescence intensity of the cells was measured using a microplate reader (Cytation 5) at 485/535 nm.

### Measurement of mitochondrial membrane potential (MMP)

MMP was measured according to a previously described method, using the fluorescent dye 5,5′,6,6′-tetrachloro-1,1′,3,3′-tetraethylbenzimidazolocarbocyanine iodide, i.e., JC-1 (Invitrogen, Thermo Fisher Scientific, Inc.)^41^. The cells treated for 24 h were rinsed with phenol red-free DMEM and incubated with 20 μM JC-1 dye in phenol red-free DMEM for 45 min at 37 °C in the dark. After washing, the cells were incubated with 20 μM carbonyl cyanide 3-chlorophenylhydrazone (CCCP; Sigma-Aldrich) for 10 min at 37 °C. The fluorescence intensity was determined in the red (Ex/Em: 550/600 nm) and green channels (Ex/Em: 485/535 nm) using a microplate reader (Cytation 5).

### Statistical analysis

All results are presented as mean ± standard error of the mean from three or more independent experiments; each experiment was performed in triplicate. Statistical analyses were performed using the SPSS software version 12 for Windows (IBM Corp., Armonk, NY, USA). Statistical significance was evaluated by one-way analysis of variance, followed by Dunnett’s *post-hoc test*; *p* < 0.05 was considered statistically significant.

## Results

### Morphological changes in SH-SY5Y/astrocyte co-cultured cells

To confirm the developmental neurological characteristics in the co-culture model, morphological changes of the co-cultured cells were observed to investigate whether they were differentiated by RA. Under a light microscope, the undifferentiated cells aggregated to form clusters on the culture plate and showed round cell bodies and short processes (Fig. 1A). Conversely, cells differentiated by RA spread regularly on the plate and developed longer neurites with a neuron-like morphology compared to undifferentiated cells (Fig. 1A). As shown in Fig. 1A, the astrocytes displayed a characteristic star shape before and after differentiation. To confirm the presence of neurons and astrocytes in the co-culture, western blotting was used to detect the following markers: nestin of the stem cell marker, microtubule-associated protein 2 (MAP2) of the neuronal differentiation marker, βIII-tubulin of the neuronal marker, and GFAP of the astrocyte marker. Differentiated cells exhibited higher levels of MAP2 and lower levels of nestin than undifferentiated cells (Fig. 1B), which is consistent with previously reported findings^42^. As shown in Fig. 1B, βIII-tubulin and GFAP were equally expressed in differentiated and undifferentiated cells. Astrocytes were not affected by RA, which is consistent with the microscopic observations. To further confirm the presence of neuronal cells and astrocytes in the co-culture, we performed double immunofluorescence staining. As shown in Fig. 1C, we validated the coexistence of neurons and astrocytes in the co-culture model. Immunostaining using anti-βIII tubulin primary antibody and Hoechst 33258 dye showed extensive neurite outgrowth in differentiated cells incubated with RA compared to that in undifferentiated cells (Fig. 1D). The average length of neurites in differentiated cells increased by approximately four-fold compared to that in undifferentiated cells (Fig. 1E). Thus, the SH-SY5Y/astrocyte co-culture model exhibited a cellular phenotype indicating the coexistence of neurons and astrocytes and a characteristic neuronal morphology in differentiated cells.

### Cytotoxic effects of CPT and ZPT on co-cultured cells

**Figure 2:**
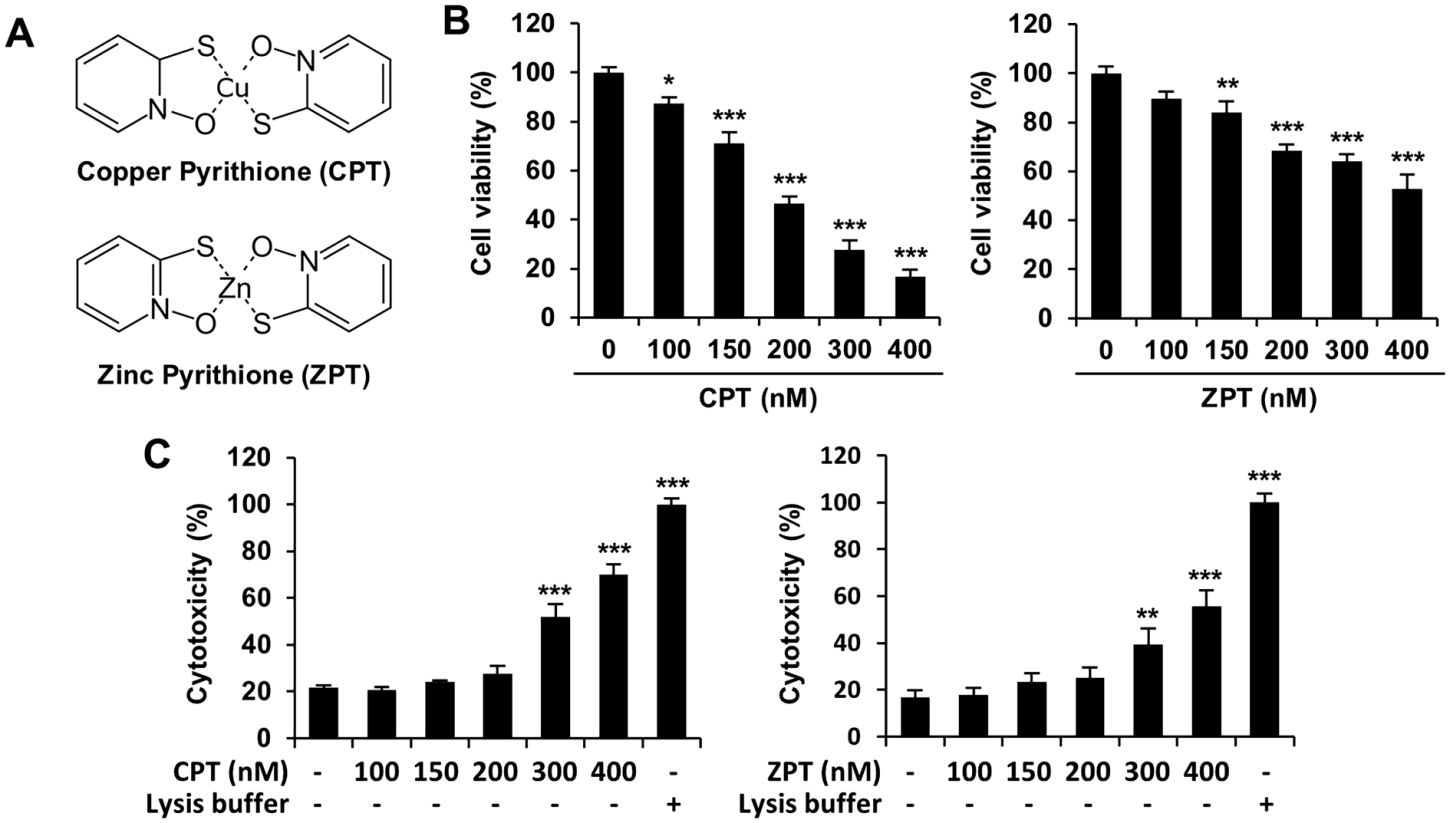
Effects of copper pyrithione (CPT) and zinc pyrithione (ZPT) on cell viability and lactate dehydrogenase (LDH) release in the co-culture model. (**A**) Structure of CPT and ZPT. (**B**) Cell viability was determined using the CellTiter 96 Aqueous One Solution Cell Proliferation Assay Kit. Data are expressed as percent of the vehicle control and are presented as means ± standard error of the mean (SEM). Results were obtained from three independent experiments. *p < 0.05, **p < 0.01, and ***p < 0.001. (**C**) Lactate dehydrogenase (LDH) release was determined by the CytoTox 96 Non-Radioactive Cytotoxicity Assay Kit. The values are shown as a percentage of lysis buffer (maximum LDH release), and data are shown as means ± SEM. Data are representative of triplicate experiments. **p < 0.01 and ***p < 0.001 compared to the control.

MTS and LDH assays were performed to evaluate the cell viability and cytotoxic effects, respectively, of CPT and ZPT (Fig. 2A) on SH-SY5Y/astrocyte co-cultured cells. A 24 h incubation period with increasing concentrations of CPT and ZPT (100–400 nM) inhibited cell viability in a dose-dependent manner compared to the control (Fig. 2B). The cells exhibited a higher sensitivity to CPT than ZPT at the same concentration. Preliminary experiments revealed IC_50_ values of 193 nM and 411 nM for CPT and ZPT, respectively. Considering that astrocytes are known to have a protective effect against neurotoxicity^31^, we further confirmed the effects of CPT and ZPT on a SH-SY5Y monoculture. In the monoculture, the cell viability inhibition rate was similar to or more sensitive than that in the co-culture (Supplementary Fig. 2A). For the SH-SY5Y monocultured cells, the IC_50_ values for CPT and ZPT were 193 nM and 272 nM, respectively. The amount of LDH released into the culture medium by cells exposed to CPT and ZPT was determined using negative (cell culture medium) and positive (cell lysis buffer) controls. The level of LDH release significantly increased to 70% and 56% after exposure to CPT and ZPT, respectively (Fig. 2C). These results suggest that CPT and ZPT showed relatively low cytotoxicity at concentrations up to 200 nM and high cytotoxicity at concentrations up to 400 nM.

### CPT and ZPT inhibit neurite outgrowth

**Figure 3:**
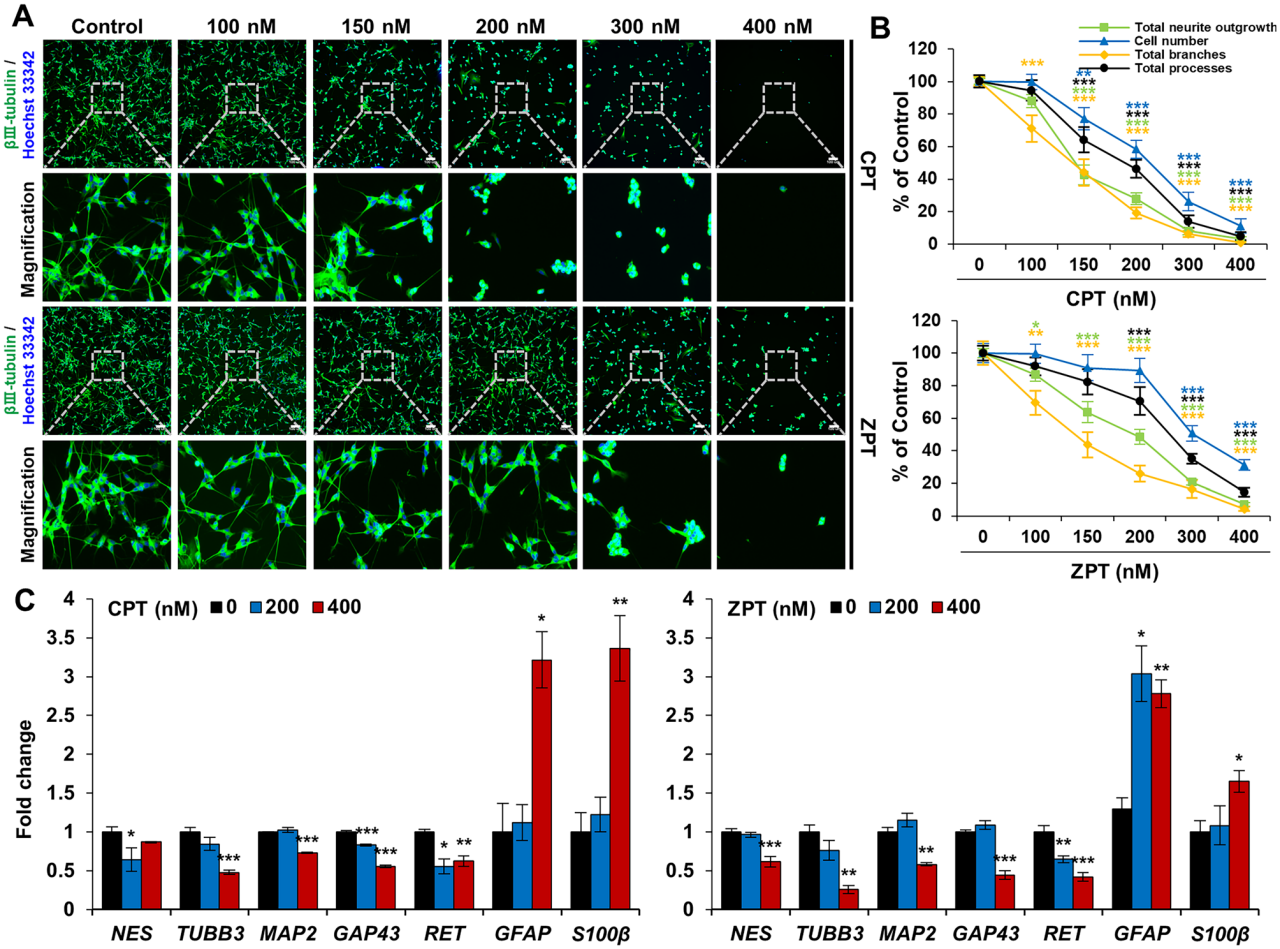
Effects of copper pyrithione (CPT) and zinc pyrithione (ZPT) exposure on neurotoxicity. SH-SY5Y and astrocyte co-cultured cells were cultured for 5 days with retinoic acid (RA) and then treated with indicated concentration of CPT and ZPT for 24 h. (**A**) Immunocytochemical images display cells stained with an anti-βIII-tubulin antibody (green) and Hoechst 33,342 (blue). Scale bar: 100 μm. (**B**) The quantified values are shown as the percentage of control and error bars represent mean ± standard error of mean (SEM). All data were obtained from three independent experiments. *p < 0.05, **p < 0.01, and ***p < 0.001 compared to the control. (**C**) Expression of *NES, TUBB3, MAP2, GAP43, RET, GFAP,* and *S100β* were determined using quantitative real-time polymerase chain reaction. The results were normalized to *β-actin* expression. Data are mean ± SEM. *p < 0.05, **p < 0.01, and ***p < 0.001.

To evaluate the neurotoxic effects of CPT and ZPT on co-cultured cells, neurite outgrowth was confirmed using immunostaining. Figure 3A shows representative immunostaining images of cells treated and untreated with CPT and ZPT. Figure 3A and 3B show that the total neurite outgrowth was significantly decreased following CPT and ZPT treatment for 24 h compared to control cells. CPT decreased the total neurite outgrowth at concentrations above 150 nM, and the number of cells also had a significant effect. ZPT affected the total neurite outgrowth at concentrations of 100 nM, whereas the number of cells decreased only at concentrations above 300 nM. In addition, as shown in Fig. 3B, CPT and ZPT significantly inhibited the total number of branches and processes in a concentration-dependent manner. Similar to what was observed in the co-culture, total neurite outgrowth, cell number, and the total number of branches and processes were significantly reduced by CPT and ZPT in the SH-SY5Y monoculture (Supplementary Fig. 2B and C). The inhibition rates in the SH-SY5Y monoculture were either more sensitive or comparable to those in the co-culture. We next assessed the changes in gene expression related to neuronal differentiation and development to determine the neurotoxic effects of CPT and ZPT (Fig. 3C). As shown in the obtained results, CPT and ZPT significantly promoted the downregulation of the neuronal differentiation and development genes *NES, TUBB3, MAP2, GAP43*, and *RET* in co-cultured cells. In contrast, at a concentration of 400 nM, CPT increased the expression levels of the astrocyte marker genes *GFAP* and *S100β* by 3.2- and 3.4-fold, respectively. For ZPT, *GFAP* and *S100β* expression levels were 2.8- and 1.7-fold higher, respectively, at a concentration of 400 nM. To gain a deeper understanding of the toxic effects of CPT and ZPT on astrocytes in relation to the expression of astrocyte marker genes, we further verified the marker gene expression for both CPT and ZPT in astrocyte monocultures. CPT was found to increase the expression of *GFAP* and *S100β* by up to 5- and 6-fold, respectively, whereas ZPT led to a 3- to 2.3-fold increase in their expression (Supplementary Fig. 3). These data suggest that CPT and ZPT induced neurotoxicity and cytotoxicity. Considering the IC_50_ values of CPT and ZPT, concentrations of 200 nM for neurotoxicity with relatively low cytotoxicity and 400 nM for neurotoxicity with high cytotoxicity were used for the subsequent experiments.

### CPT and ZPT induce ROS production and disrupt the MMP

**Figure 4:**
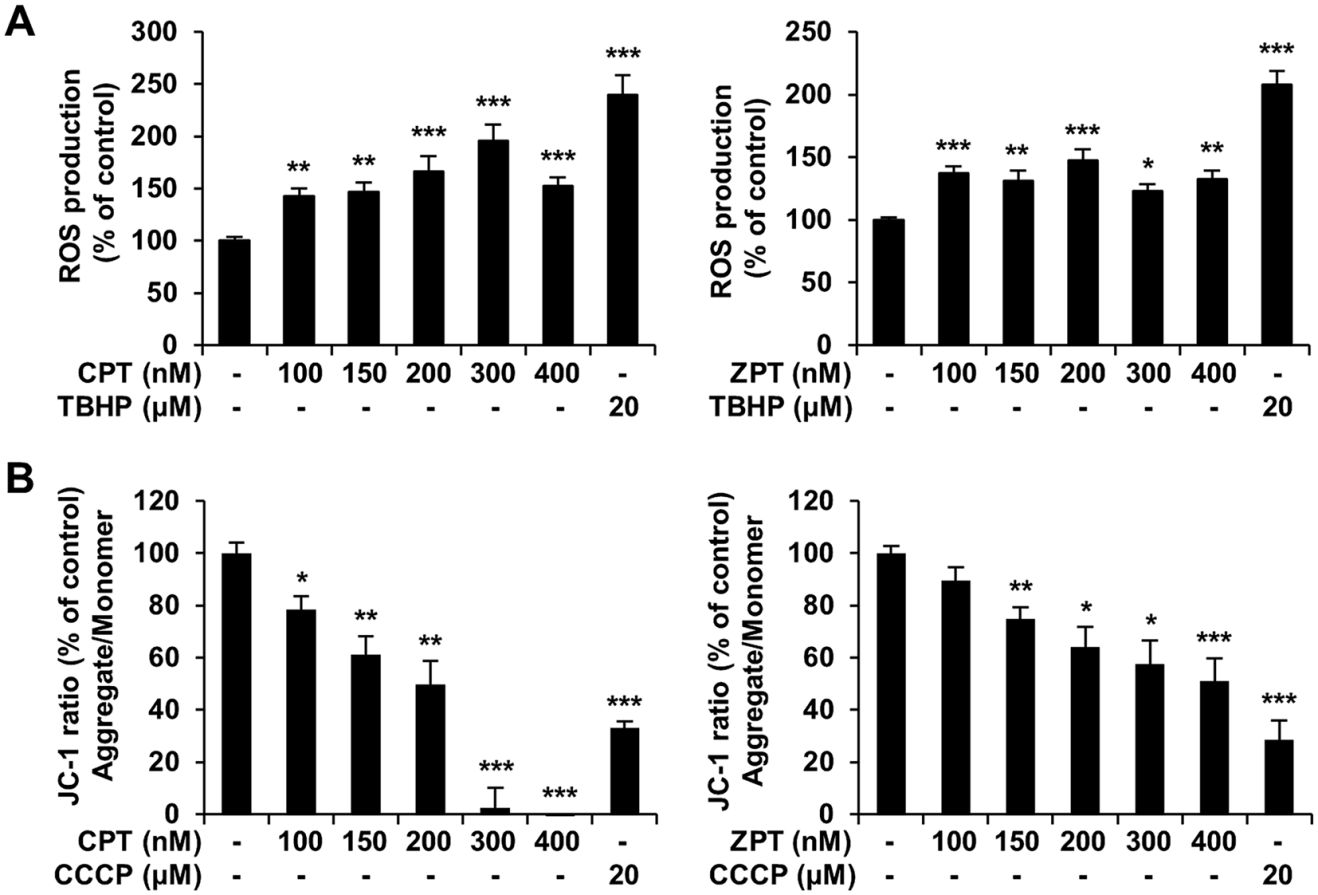
Effects of copper pyrithione (CPT) and zinc pyrithione (ZPT) on reactive oxygen species (ROS) generation and mitochondrial function in co-cultured cells. Co-cultured cells were incubated with CPT and ZPT at the indicated concentration for 24 h. (**A**) Intracellular ROS were measured with a microplate reader using DCF-DA fluorescent dye. Cells treated for 1 h with 20 μM tert-Butyl hydroperoxide solution (TBHP) served as a positive control. Results of at least three independent experiments are presented as the mean ± standard error of mean (SEM). *p < 0.05, **p < 0.01, and ***p < 0.001 versus control. (**B**) Mitochondrial membrane potential (MMP) was determined using a fluorescence probe, JC-1, as described in Section “Material and methods”. Cells treated for 10 min with 20 μM carbonyl cyanide 3-chlorophenylhydrazone (CCCP) served as a positive control. The results were presented as mean ± SEM. *p < 0.05, **p < 0.01, and ***p < 0.001 versus control.

To investigate whether CPT and ZPT stimulated ROS generation in SH-SY5Y/astrocyte co-cultured cells, intracellular ROS were analyzed by fluorescence microscopy after staining with the DCFDA reagent. As shown in Fig. 4A, CPT and ZPT induced the accumulation of ROS; the intracellular ROS levels increased by up to twofold after CPT treatment and up to 1.5-fold after ZPT treatment compared to the control group. To evaluate the effect of CPT and ZPT on mitochondrial function, the MMP was monitored using a fluorescent JC-1 dye, and the JC-1 red/green fluorescence intensity ratio was calculated. Exposure of cells to CPT and ZPT for 24 h disrupted the MMP, which was evidenced by a decrease in the red/green ratio of JC-1 fluorescence, as shown in the results of the positive control (20 μM CCCP; Fig. 4B). Given that the observed ROS levels and JC-1 results at the 24 h mark following exposure to CPT and ZPT may be associated with cell death, as indicated by the results of the cell viability analysis, we reassessed ROS production and JC-1 levels at an earlier time point (6 h). After a 6 h exposure to CPT and ZPT, no significant effect on ROS levels was observed at any concentration (Supplementary Fig. 4A). The analysis of JC-1 confirmed significant inhibition at 300 and 400 nM for CPT, and only at 400 nM for ZPT (Supplementary Fig. 4B). These results demonstrate that CPT and ZPT have the potential to cause ROS generation and mitochondrial dysfunction.

### NAC prevents CPT- and ZPT-induced cell growth and neurite outgrowth inhibition in co-cultured cells

**Figure 5:**
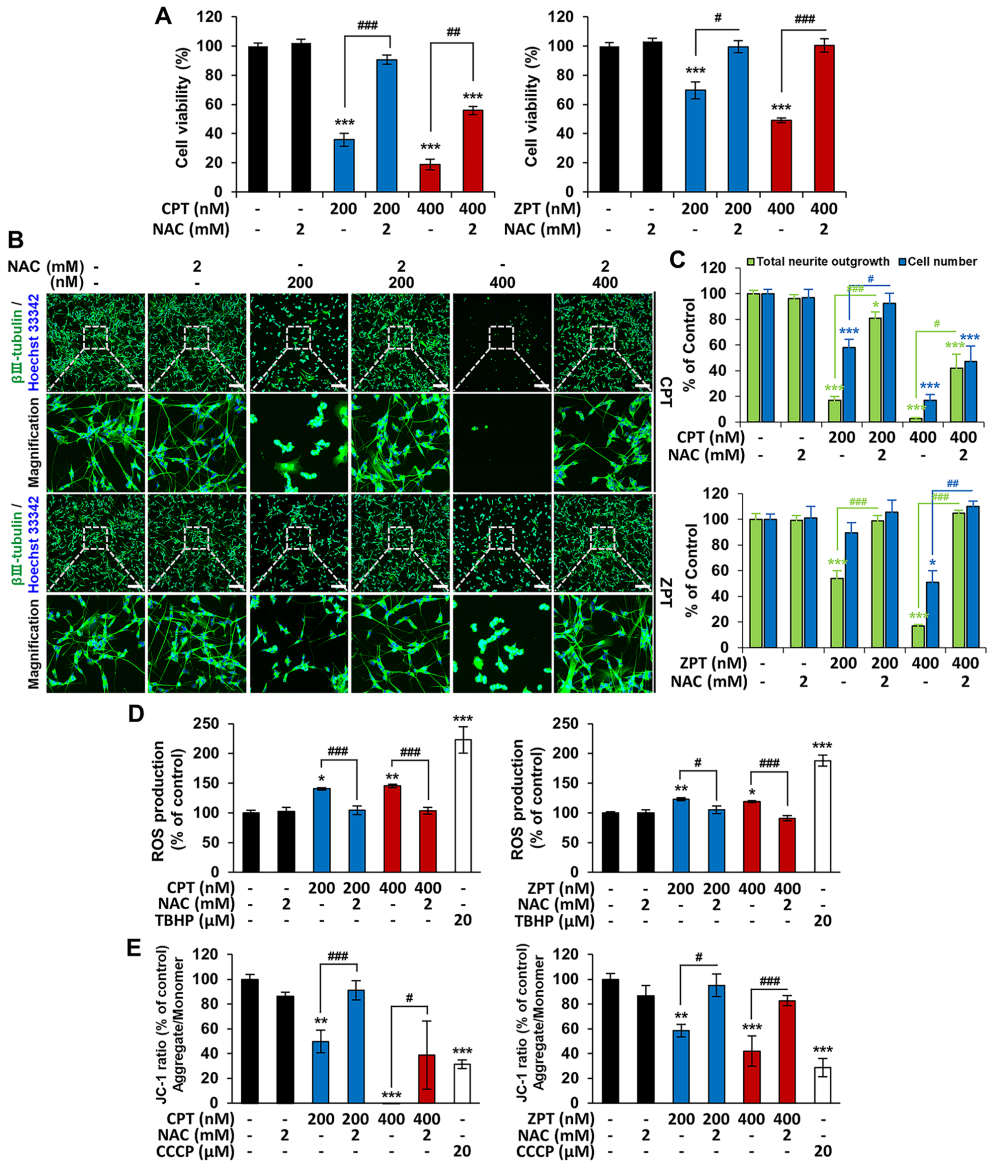
Protective effect of* N*-acetylcysteine (NAC) against copper pyrithione (CPT) and zinc pyrithione (ZPT) toxicity in co-cultured cells. The cells were pretreated with NAC at 2 mM for 1 h followed by incubation with CPT and ZPT for 24 h. (**A**) Cell viability was determined using the CellTiter 96 Aqueous One Solution cell Proliferation Assay Kit. Data of at least three independent experiments are presented as the mean ± SEM. (**B**) Representative fluorescent images of cells stained with an anti-βIII-tubulin antibody (green) and Hoechst 33342 (blue). Scale bar: 200 μm. (**C**) Data of triplicates are shown as mean ± SEM. (**D**) The DCF-DA fluorescence intensity was measured using a microplate reader. Error bars represent the mean ± SEM of three independent experiments. (**E**) Disruption of the mitochondrial membrane potential (MMP) was detected by JC-1 staining and measured using a microplate reader. The graph was expressed as a percentage compared to the control group, and carbonyl cyanide 3-chlorophenylhydrazone (CCCP) was used for the positive control group. Data are representative of at least three independent experiments. *p < 0.05, **p < 0.01 and ***p < 0.001 compared to the control group. ^#^*p* < 0.05, ^##^*p* < 0.01, and ^###^*p* < 0.001 compared to the CPT- or ZPT-treated group.

To determine whether the cytotoxicity and neurotoxicity induced by CPT and ZPT depended on ROS generation, we analyzed their effects on cell viability and neurite outgrowth in the absence and presence of the antioxidant NAC. As shown in Fig. 5A, cell viability significantly decreased after treatment with CPT and ZPT (200 and 400 nM), and this effect was inhibited by NAC. NAC pretreatment significantly inhibited CPT- and ZPT-induced reduction in total neurite outgrowth and cell number compared with the untreated cells (Fig. 5B and C). These data suggest that ROS is associated with neuronal survival.

### NAC prevents the generation of ROS and loss of MMP by CPT and ZPT

Subsequently, NAC was used to evaluate the role of ROS and MMP in the toxic effects CPT and ZPT. As shown in Fig. 5D, ROS levels significantly increased after treatment with 200 or 400 nM CPT and ZPT, and this effect was prevented by the ROS scavenger NAC. Exposure of human SH-SY5Y/astrocyte cells to CPT and ZPT (200 or 400 nM) for 24 h disrupted the MMP, as demonstrated by a decrease in the proportion of cells with a red/green ratio of JC-1 (Fig. 5E). Pretreatment with NAC effectively prevented the disruption of the MMP by CPT and ZPT (Fig. 5E). These data suggest that NAC inhibits the mitochondrial dysfunction and ROS generation induced by CPT and ZPT.

## Discussion

Cu and Zn are essential trace metal ions in the CNS and play a variety of biological functions^43,44^. Since Cu and Zn are involved in the functioning of various enzymes, their deficiency or excess can lead to neurotoxicity^44,45^. Because antifouling agents, CPT and ZPT can interact with free metal ions in seawater by releasing Cu and Zn metal ions^13^. The pyrithiones in CPT and ZPT are known to function as ionophores of Cu^2+^ and Zn^2+^ ions, respectively^13,18^. Studies have indicated that pyrithione (sodium pyrithione) and Zn^2+^ individually demonstrated non-toxicity to cells^18^. Simultaneous treatment with both, however, resulted in cytotoxicity that is similar to ZPT^18^. This suggests that CPT and ZPT may induce cytotoxicity and neurotoxicity because pyrithione acts as a Cu^2+^ and Zn^2+^ ionophore, resulting in an increase in the level of free metal ions in cells. ZPT is known to increase intracellular Zn^2+^ concentrations and is potentially associated with oxidative stress^17^. In the nervous system, oxidative stress is induced by several internal and external factors; excessive ROS levels can mediate cytotoxicity and is intrinsically linked with mitochondrial dysfunction^33,46^. Exposure to Cu and Zn ions has been shown to induce mitochondrial depolarization and ROS production^18,47,48^. Therefore, we hypothesized that the neurotoxic effects induced by CPT and ZPT are due to oxidative stress. Exposure to CPT and ZPT resulted in mitochondrial dysfunction and intracellular ROS generation (Fig. 4). Although there is limited evidence on the relationship between CPT and oxidative stress, recent studies have shown that CPT induces the production of ROS, resulting in oxidative stress in the hemocytes of *Litopenaeus vannamei*^49^. Although some studies have reported that ZPT exposure has no effect on ROS production, others have reported that it induces ROS production. It is likely that these studies have shown no effect on oxidative stress because ZPT was only applied for a short period of time, such as 30 min or 6 h^50^. These findings were consistent with the results obtained in the present study after a 6 h short-term exposure to CPT and ZPT (Supplementary Fig. 4A). In contrast, another study that used the same exposure time adopted here (24 h) reported a slight increase in ROS generation induced by ZPT, which is consistent with our findings^18,51^. Since ROS levels showed only a slight increase by ZPT compared to CPT, and several studies have reported conflicting results, further experiments may be needed to confirm the association between ZPT and ROS. In addition, since the in vitro experiments cannot fully reflect ROS generation in vivo, measuring ROS through in vivo experiments may provide more accurate results.

NAC is a widely known ROS scavenger and is currently emerging as a treatment for neurological disorders^35^. NAC is a precursor of l-cysteine and reduced glutathione; it exhibits antioxidant effects by reacting with ROS or inducing glutathione production^35^. In the nervous system, NAC enhances neuronal differentiation and neuritogenesis, which may be utilized as a therapeutic strategy for targeting neuron loss or neurite dystrophy^36^. For example, NAC shows protective effects against insecticide-induced oxidative stress-related cell death in normal human astrocytes^37^. Here, NAC showed a protective effect against CPT- and ZPT-mediated cytotoxicity and neurotoxicity regardless of their concentrations (Fig. 5). NAC also attenuated CPT- and ZPT-mediated oxidative stress and mitochondrial dysfunction (Fig. 5). Thus, it appears that pretreatment with NAC provided an antioxidant effect in CPT- and ZPT-treated SH-SY5Y/astrocyte co-cultured cells. However, in addition to its antioxidant effects, NAC possesses metal-chelating properties and has been demonstrated to regulate Cu and Zn homeostasis^52^. Consequently, definitively attributing the cytoprotective effect of NAC solely to the chelation of free metal ions or its antioxidant properties is challenging. This ambiguity could be addressed through additional experiments using alternative antioxidants that lack metal-chelating properties, further elucidating the role of oxidative stress in the toxicity of CPT and ZPT. Taken together, these results suggest that oxidative stress may be involved in the cytotoxicity and neurotoxicity induced by CPT and ZPT.

As end-point assays, such as that of oxidative stress, do not differentiate between neurotoxicity and cytotoxicity, a neuron-specific assay is also needed. Neurotoxicity can occur at lower concentrations that do not affect neuronal survival^53^. In particular, CPT and ZPT induced neurotoxicity at non-cytotoxic concentrations, as demonstrated by the neurite outgrowth assay and qRT-PCR analysis (Fig. 3). However, despite testing the neurotoxicity and cytotoxicity of CPT and ZPT using assays that evaluate various end-point in cell systems, no clear distinction has been made between neurotoxicants and cytotoxicants. As neurotoxicity and cytotoxicity are closely related, attention should be directed towards the toxic effects of CPT and ZPT. Exposure to CPT and ZPT has been shown to have an effect on neurites by similarly reducing the total neurite length as well as other parameters that represent neurite complexity. In addition to neurite analysis, the altered expression of genes associated with neurodevelopment may reinforce evidence of neurotoxicity. Neuronal differentiation decreases the levels of nestin, whereas astrocytic differentiation increases GFAP levels^54^. βIII-tubulin, a microtubule element, has been shown to be consistently expressed in differentiated and undifferentiated neurons^55^. MAP2 is a major microtubule-associated brain protein that is selectively localized in dendrites, whereas GAP43 is a neuron-specific protein associated with axonal outgrowth^56^. They are specifically localized in mature neurons and are expressed at all stages of neuronal development^57^. The receptor tyrosine kinase RET is known to be expressed in most neuroblastoma cell lines and is required for neurodevelopment^58^. In this study, well-known markers of neuronal development and maturation were downregulated by CPT and ZPT, suggesting that CPT and ZPT induce neurotoxicity via pathways related to neurodevelopment and maturation (Fig. 3C). Conversely, astrocyte markers were upregulated, indicating neurodegeneration and injury^59^. These results show that CPT and ZPT affect not only neurons, but also astrocytes, thereby causing nerve damage. Therefore, the altered expression of neural and astrocyte-related genes in co-culture models indicates the possibility of neurotoxicity induced by exposure to CPT and ZPT.

A relatively large number of studies on ZPT-mediated neurotoxicity and cytotoxicity have been conducted, whereas similar studies on CPT are limited. This study is the first to report on the neurotoxic effect of CPT, and our results indicate that it is even more toxic than ZPT. Intracellular Zn^2+^ accumulation by ZPT induced cell death in differentiated PC12 neuron cells, which demonstrated the cytotoxic and neurotoxic potential of ZPT^60^. According to the available toxicology data, ZPT is considered safe at concentrations of up to 2% w/v^61^; however, it should be noted that neurotoxicity has occurred at significantly lower concentrations. In addition, the potential risk of toxicity from exposure to CPT and ZPT is high because it depends on a variety of factors (e.g., dose, frequency, formulation, etc.). ZPT induced cytotoxicity at nanomolar to micromolar concentrations in various cell lines, such as human skin fibroblasts, prostate cancer cells, and liver cancer cells^16,18,62^. ZPT was not cytotoxic in rat thymocytes at micromolar concentrations, but enhanced H_2_O_2_ toxicity at nanomolar concentrations^17^. To our knowledge, there are no in vitro studies that have evaluated the cytotoxic effects of CPT on human cells; however, CPT has been shown to be cytotoxic in some fish cells. In suspension-cultured fish cells, the 24 h EC_50_ values of CPT and ZPT were 0.1 mg/L (316.6 nM) and 0.18 mg/L (566.6 nM), respectively, and in vivo experiments using rainbow trout also showed that CPT was more toxic than ZPT^14^. The cytotoxicity of CPT and ZPT in neuron/astrocyte co-cultured cell models in this study is similar to the results reported in several previous studies (Fig. 2). Therefore, further studies using other concentrations are required to define the relationship between neurotoxicity and cytotoxicity. Finally, because only short-term exposure (24 h) was evaluated here, the toxic effects of long-term exposure should also be considered.

## Conclusion

In this study, we confirmed the neurotoxic and cytotoxic potential of CPT and ZPT in neuron and astrocyte co-culture cell models and explored the underlying cellular mechanisms. Comprehensive data from human SH-SY5Y/astrocyte co-cultured cells demonstrate that CPT and ZPT induce cytotoxicity and neurotoxicity by altering the expression of neurodevelopmental genes, inhibiting neurite outgrowth, and inducting oxidative stress and mitochondrial dysfunction. Hence, approaches using the antioxidant NAC offer an efficient strategy to counteract the toxic effects of CPT and ZPT on the human brain. Our findings provide an insight into the toxicological mechanisms of CPT and ZPT and can serve as a fundamental basis for the use and handling of these agents.

### Supplementary Information

Supplementary Figures.

## Data Availability

The datasets used during the current study available from the corresponding author on reasonable request.

## Abbreviations

BBB: Blood-brain barrier
CPT: Copper pyrithione
ZPT: Zinc pyrithione
RA: Retinoic acid
ROS: Reactive oxygen species
NAC: *N*-acetylcysteine
DMEM: Dulbecco's modified Eagle's medium
FBS: Fetal bovine serum
P/S: Penicillin/streptomycin
PBS: Phosphate-buffered saline
DMSO: Dimethyl sulfoxide
LDH: Lactate dehydrogenase
RT: Room temperature
qRT-PCR: Quantitative real-time polymerase chain reaction
MMP: Mitochondrial membrane potential
MAP2: Microtubule-associated protein 2
GFAP: Glial fibrillary acidic protein
TBHP: tert-Butyl hydroperoxide
CCCP: Carbonyl cyanide 3-chlorophenylhydrazone

## References

[1] Amara I, Miled W, Slama RB, Ladhari N (2018). Antifouling processes and toxicity effects of antifouling paints on marine environment. A review. Environ. Toxicol. Pharmacol..

[2] Qian PY, Chen L, Xu Y (2013). Mini-review: molecular mechanisms of antifouling compounds. Biofouling.

[3] Kim S, Seo M, Na M, Kim J (2021). Investigation on combined inhalation exposure scenarios to biocidal mixtures: biocidal and household chemical products in South Korea. Toxics.

[4] Schwartz JR (2016). Zinc Pyrithione: A topical antimicrobial with complex pharmaceutics. J. Drugs Dermatol..

[5] Union, E. *Copper Pyrithione Product Type 21* 88, (2015).

[6] Holmes AM, Kempson I, Turnbull T, Paterson D, Roberts MS (2018). Imaging the penetration and distribution of zinc and zinc species after topical application of zinc Pyrithione to human skin. Toxicol. Appl. Pharmacol..

[7] Shin HK (2021). Development of blood brain barrier permeation prediction models for organic and inorganic biocidal active substances. Chemosphere.

[8] Daina A, Michielin O, Zoete V (2017). SwissADME: A free web tool to evaluate pharmacokinetics, drug-likeness and medicinal chemistry friendliness of small molecules. Sci. Rep..

[9] Kladnik J (2022). Zinc Pyrithione is a potent inhibitor of PL^Pro^ and cathepsin L enzymes with ex vivo inhibition of SARS-CoV-2 entry and replication. J. Enzyme Inhib. Med. Chem..

[10] Mochida K (2009). Inhibition of acetylcholinesterase by metabolites of copper Pyrithione (CuPT) and its possible involvement in vertebral deformity of a CuPT-exposed marine teleostean fish. Comp. Biochem. Physiol. C Toxicol. Pharmacol..

[11] Jeffcoat AR (1980). Zinc pyridinethione: urinary metabolites of zinc pyridinethione in rabbits, rats, monkeys, and dogs after oral dosing. Toxicol. Appl. Pharmacol..

[12] Sahenk Z, Mendell JR (1979). Ultrastructural study of zinc pyridinethione-induced peripheral neuropathy. J. Neuropathol. Exp. Neurol..

[13] Maraldo K, Dahllöf I (2004). Indirect estimation of degradation time for zinc Pyrithione and copper Pyrithione in seawater. Mar. Pollut. Bull..

[14] Okamura H, Watanabe T, Aoyama I, Hasobe M (2002). Toxicity evaluation of new antifouling compounds using suspension-cultured fish cells. Chemosphere.

[15] Mochida K, Amano H, Onduka T, Kakuno A, Fujii K (2011). Toxicity and metabolism of copper Pyrithione and its degradation product, 2,2′-dipyridyldisulfide in a marine polychaete. Chemosphere.

[16] Rudolf E, Cervinka M (2011). Stress responses of human dermal fibroblasts exposed to zinc Pyrithione. Toxicol. Lett..

[17] Oyama TM, Saito M, Yonezawa T, Okano Y, Oyama Y (2012). Nanomolar concentrations of zinc Pyrithione increase cell susceptibility to oxidative stress induced by hydrogen peroxide in rat thymocytes. Chemosphere.

[18] Mo J, Lin D, Wang J, Li P, Liu W (2018). Apoptosis in HepG2 cells induced by zinc Pyrithione via mitochondrial dysfunction pathway: involvement of zinc accumulation and oxidative stress. Ecotoxicol. Environ. Saf..

[19] Mann JJ, Fraker PJ (2005). Zinc Pyrithione induces apoptosis and increases expression of Bim. Apoptosis.

[20] EPA. *Guidelines for Neurotoxicity Risk Assessment* (1998).

[21] Cheung YT (2009). Effects of all-trans-retinoic acid on human SH-SY5Y neuroblastoma as in vitro model in neurotoxicity research. Neurotoxicology.

[22] Kovalevich J, Langford D (2013). Considerations for the use of SH-SY5Y neuroblastoma cells in neurobiology. Methods Mol. Biol..

[23] Radio NM, Mundy WR (2008). Developmental neurotoxicity testing in vitro: models for assessing chemical effects on neurite outgrowth. Neurotoxicology.

[24] Anderl JL, Redpath S, Ball AJ (2009). A neuronal and astrocyte co-culture assay for high content analysis of neurotoxicity. J. Vis. Exp..

[25] Oh HN (2022). In vitro neurotoxicity evaluation of biocidal disinfectants in a human neuron-astrocyte co-culture model. Toxicol. Vitro.

[26] Farhy-Tselnicker I, Allen NJ (2018). Astrocytes, neurons, synapses: a tripartite view on cortical circuit development. Neural Dev..

[27] Batenburg KL (2023). A 3D human co-culture to model neuron-astrocyte interactions in tauopathies. Biol. Proced. Online.

[28] Goshi N, Kim H, Girardi G, Gardner A, Seker E (2023). Electrophysiological activity of primary cortical neuron-glia mixed cultures. Cells.

[29] Yu PH, Zuo DM (1997). Enhanced tolerance of neuroblastoma cells towards the neurotoxin 6-hydroxydopamine following specific cell-cell interaction with primary astrocytes. Neuroscience.

[30] Angelova PR, Myers I, Abramov AY (2023). Carbon monoxide neurotoxicity is triggered by oxidative stress induced by ROS production from three distinct cellular sources. Redox Biol..

[31] Bélanger M, Magistretti PJ (2009). The role of astroglia in neuroprotection. Dial. Clin. Neurosci..

[32] Salim S (2017). Oxidative stress and the central nervous system. J. Pharmacol. Exp. Ther..

[33] Sayre LM, Perry G, Smith MA (2008). Oxidative stress and neurotoxicity. Chem. Res. Toxicol..

[34] Nicholls DG, Budd SL (2000). Mitochondria and neuronal survival. Physiol. Rev..

[35] Bavarsad Shahripour R, Harrigan MR, Alexandrov AV (2014). N-acetylcysteine (NAC) in neurological disorders: mechanisms of action and therapeutic opportunities. Brain Behav..

[36] Qian HR, Yang Y (2009). Neuron differentiation and neuritogenesis stimulated by N-acetylcysteine (NAC). Acta Pharmacol. Sin..

[37] Shieh P, Jan CR, Liang WZ (2019). The protective effects of the antioxidant N-acetylcysteine (NAC) against oxidative stress-associated apoptosis evoked by the organophosphorus insecticide Malathion in normal human astrocytes. Toxicology.

[38] Soleimani Asl S, Saifi B, Sakhaie A, Zargooshnia S, Mehdizadeh M (2015). Attenuation of ecstasy-induced neurotoxicity by N-acetylcysteine. Metab. Brain Dis..

[39] Monti DA (2016). N-acetyl cysteine may support dopamine neurons in Parkinson's disease: Preliminary clinical and cell line data. PLOS ONE.

[40] Livak KJ, Schmittgen TD (2001). Analysis of relative gene expression data using real-time quantitative PCR and the 2-ΔΔCT Method. Methods.

[41] Sivandzade F, Bhalerao A, Cucullo L (2019). Analysis of the mitochondrial membrane potential using the cationic JC-1 dye as a sensitive fluorescent probe. Bio Protoc..

[42] Murillo JR (2017). Quantitative proteomic analysis identifies proteins and pathways related to neuronal development in differentiated SH-SY5Y neuroblastoma cells. EuPA Open Proteomics.

[43] Opazo CM, Greenough MA, Bush AI (2014). Copper: from neurotransmission to neuroproteostasis. Front. Aging Neurosci..

[44] Choi S, Hong DK, Choi BY, Suh SW (2020). Zinc in the brain: Friend or foe?. Int. J. Mol. Sci..

[45] Kardos J (2018). Copper signalling: Causes and consequences. Cell Commun. Signal.

[46] Wang X, Michaelis EK (2010). Selective neuronal vulnerability to oxidative stress in the brain. Front. Aging Neurosci..

[47] Weiss JH, Sensi SL, Koh JY (2000). Zn^2+^: A novel ionic mediator of neural injury in brain disease. Trends Pharmacol. Sci..

[48] Gyulkhandanyan AV, Feeney CJ, Pennefather PS (2003). Modulation of mitochondrial membrane potential and reactive oxygen species production by copper in astrocytes. J. Neurochem..

[49] Chen T, Li S, Liang Z, Li L, Guo H (2022). Effects of copper Pyrithione (CuPT) on apoptosis, ROS production, and gene expression in hemocytes of white shrimp litopenaeus vannamei. Comp. Biochem. Physiol. C Toxicol. Pharmacol..

[50] Lamore SD, Cabello CM, Wondrak GT (2010). The topical antimicrobial zinc Pyrithione is a heat shock response inducer that causes DNA damage and PARP-dependent energy crisis in human skin cells. Cell Stress Chaperones.

[51] Rudolf E, Červinka M (2010). Zinc Pyrithione induces cellular stress signaling and apoptosis in Hep-2 cervical tumor cells: the role of mitochondria and lysosomes. Biometals.

[52] Wolfram T (2020). N-acetylcysteine as modulator of the essential trace elements copper and zinc. Antioxidants.

[53] Radio NM, Breier JM, Shafer TJ, Mundy WR (2008). Assessment of chemical effects on neurite outgrowth in PC12 cells using high content screening. Toxicol. Sci..

[54] Rieske P, Azizi SA, Augelli B, Gaughan J, Krynska B (2007). A population of human brain parenchymal cells express markers of glial, neuronal and early neural cells and differentiate into cells of neuronal and glial lineages. Eur. J. Neurosci..

[55] Guo J, Walss-Bass C, Ludueña RF (2010). The β isotypes of tubulin in neuronal differentiation. Cytoskeleton.

[56] Triarhou LC, Solà C, Palacios JM, Mengod G (1998). MAP2 and GAP-43 expression in normal and weaver mouse cerebellum: Correlative immunohistochemical and in situ hybridization studies. Arch. Histol. Cytol..

[57] Kleiman R, Banker G, Steward O (1994). Development of subcellular mRNA compartmentation in hippocampal neurons in culture. J. Neurosci..

[58] Futami H, Sakai R (2009). RET protein promotes non-adherent growth of NB-39-nu neuroblastoma cell line. Cancer Sci..

[59] Jurga AM, Paleczna M, Kadluczka J, Kuter KZ (2021). Beyond the GFAP-astrocyte protein markers in the brain. Biomolecules.

[60] Seo SR (2001). Zn2+-induced ERK activation mediated by reactive oxygen species causes cell death in differentiated PC12 cells. J. Neurochem..

[61] Mangion SE, Holmes AM, Roberts MS (2021). Targeted delivery of zinc Pyrithione to skin epithelia. Int. J. Mol. Sci..

[62] Carraway RE, Dobner PR (2012). Zinc Pyrithione induces ERK- and PKC-dependent necrosis distinct from TPEN-induced apoptosis in prostate cancer cells. Biochim. Biophys. Acta.

