# Supplementary material for: Copper pyrithione and zinc pyrithione induce cytotoxicity and neurotoxicity in neuronal/astrocytic co-cultured cells via oxidative stress
Source: Sci Rep. 2023 Dec 27;13:23060. doi: 10.1038/s41598-023-49740-8 (PMC10754844; doi:10.1038/s41598-023-49740-8)
Supplement: Supplementary file 1 — Supplementary Figures. [file 41598_2023_49740_MOESM1_ESM.pdf]

[Supplementary Information]

Copper pyrithione and zinc pyrithione induce cytotoxicity and neurotoxicity in neuronal/astrocytic co-cultured cells via oxidative stress

Ha-Na Oh[a], Woo-Keun Kim[a,b,*]

[a] Department of Predictive Toxicology, Korea Institute of Toxicology, Daejeon 34114, Republic of Korea

[b] Human and Environmental Toxicology, University of Science and Technology, Daejeon, 34113, Republic of Korea

[Supplementary Figure 1]

Uncropped image in Fig. 1B

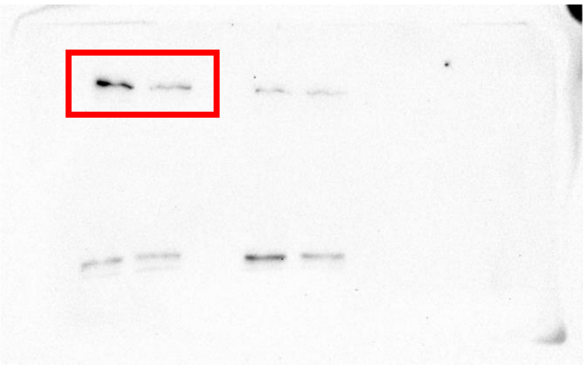

← Nestin

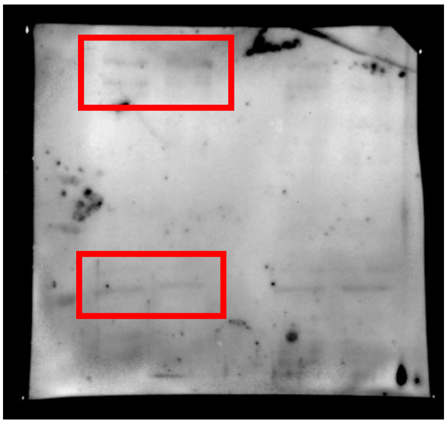

← MAP2 a/b and MAP2 c

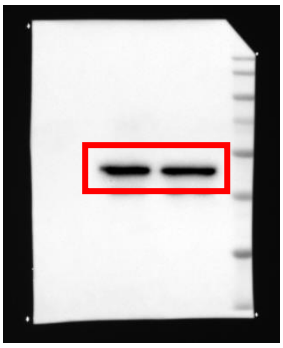

← βIII-tubulin

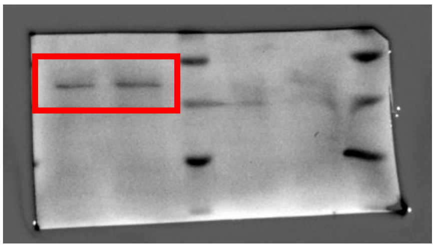

← GFAP

[Supplementary Figure 2]

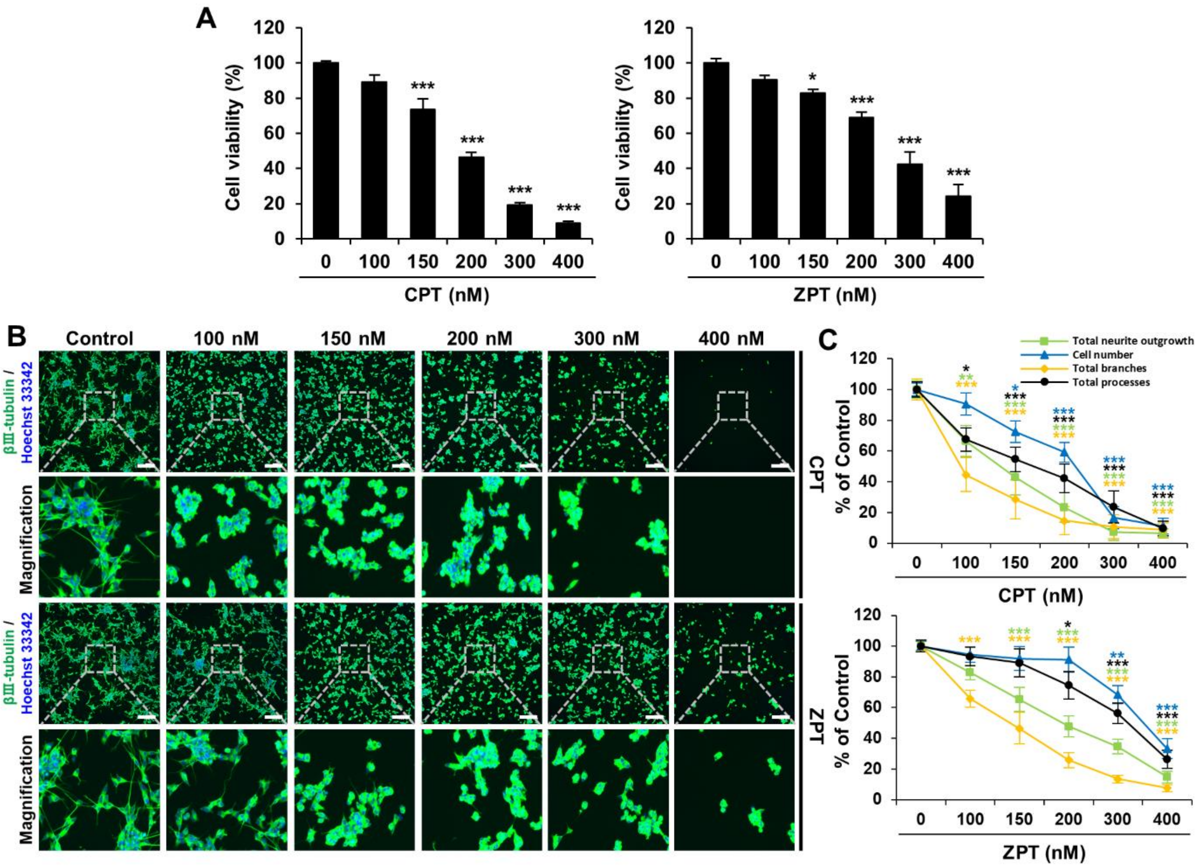

Fig. 2. Effects of copper pyrithione (CPT) and zinc pyrithione (ZPT) on cytotoxicity and neurotoxicity in SH-SY5Y cells.

(A) SH-SY5Y cells were treated with different concentrations of CPT and ZPT for 24 h. Cell viability was determined using the CellTiter 96 Aqueous One Solution Cell Proliferation Assay Kit. Results were presented as the mean ± SEM. *$p < 0.05$ and ***$p < 0.001$. (B and C) Neurite outgrowth parameters including total neurite outgrowth (green), cell number (blue), total branches (yellow), and total processes (black) were quantified after CPT and ZPT treatment. Scale bar: 200 µm. Results were presented as the mean ± SEM. *$p < 0.05$, **$p < 0.01$, and ***$p < 0.001$.

[Supplementary Figure 3]

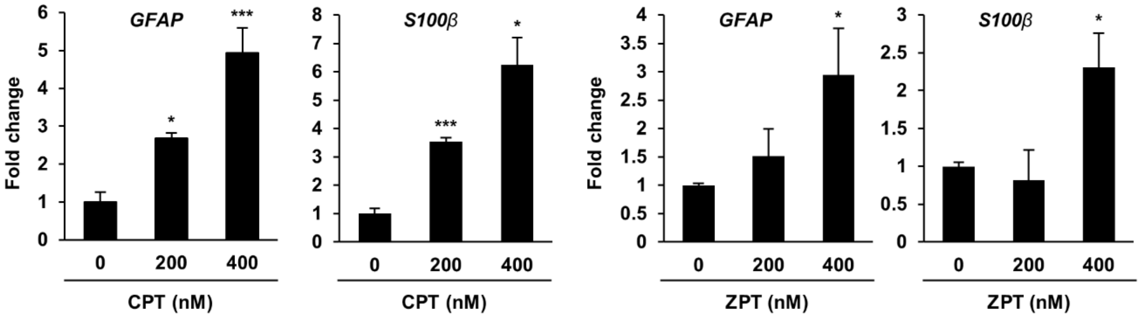

Fig. 3. Effects of copper pyrithione (CPT) and zinc pyrithione (ZPT) on neurodevelopment-related gene in astrocytes.

Astrocytes were treated with indicated concentrations of CPT and ZPT for 24 h. Expression of *GFAP* and *S100* was determined by quantitative real-time PCR. Results were presented as the mean ± SEM. *$p < 0.05$, and ***$p < 0.001$.

[Supplementary Figure 4]

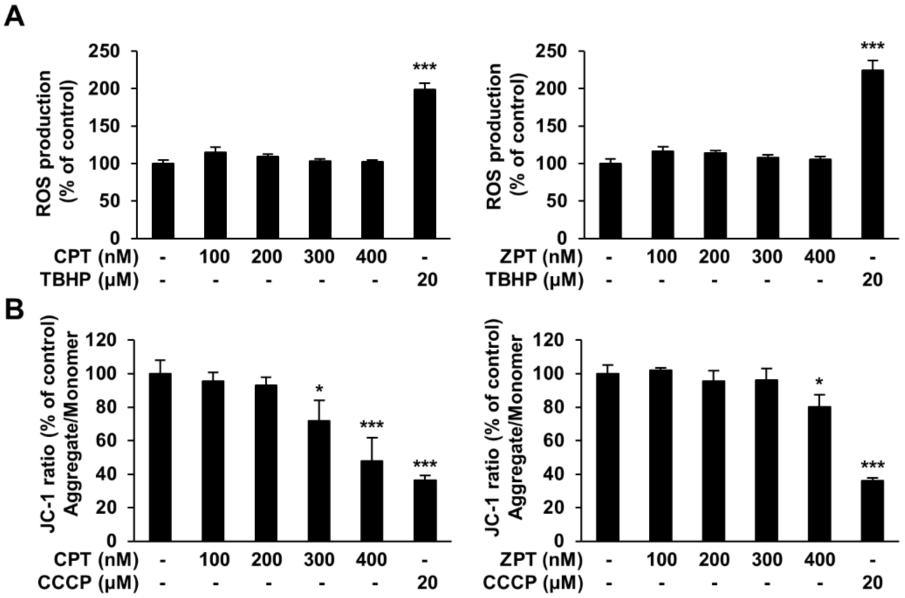

Fig. 4. Effects of copper pyrithione (CPT) and zinc pyrithione (ZPT) on reactive oxygen species (ROS) generation and mitochondrial function in co-cultured cells.

Co-cultured cells were treated with different concentrations of CPT and ZPT for 6 h. (A) ROS generation was determined using DCF-DA fluorescent dye. Results were presented as the mean ± SEM. ***$p < 0.001$. (B) Mitochondrial membrane potential (MMP) was measured using JC-1 Fluorescence probe. Results were presented as the mean ± SEM. *$p < 0.05$, and ***$p < 0.001$.
